# Italian network for obesity and cardiovascular disease surveillance: A pilot project

**DOI:** 10.1186/1471-2296-9-53

**Published:** 2008-09-29

**Authors:** Chiara Donfrancesco, Cinzia Lo Noce, Ovidio Brignoli, Gabriele Riccardi, Paola Ciccarelli, Francesco Dima, Luigi Palmieri, Simona Giampaoli

**Affiliations:** 1 Centro Nazionale di Epidemiologia, Sorveglianza e Promozione della Salute, Istituto Superiore di Sanità, Rome, Italy; 2 Società Italiana di Medicina Generale, Rome, Italy; 3 Università Federico II, Naples, Italy

## Abstract

**Background:**

Also in Mediterranean countries, which are considered a low risk population for cardiovascular disease (CVD), the increase in body mass index (BMI) has become a public health priority. To evaluate the feasibility of a CVD and obesity surveillance network, forty General Practitioners (GPs) were engaged to perform a screening to assess obesity, cardiovascular risk, lifestyle habits and medication use.

**Methods:**

A total of 1,046 women and 1,044 men aged 35–74 years were randomly selected from GPs' lists stratifying by age decade and gender. Anthropometric and blood pressure measurements were performed by GPs using standardized methodologies. BMI was computed and categorized in normal weight (BMI 18.5–24.9 kg/m^2^), overweight (BMI 25.0–29.9 kg/m^2^) and obese (BMI ≥ 30 kg/m^2^). Food frequency (per day: fruits and vegetables; per week: meat, cheese, fish, pulses, chocolate, fried food, sweet, wholemeal food, rotisserie food and sugar drink) and physical activity (at work and during leisure time) were investigated through a questionnaire. CVD risk was assessed using the Italian CUORE Project risk function.

**Results:**

The percentage of missing values was very low. Prevalence of overweight was 34% in women and 50% in men; prevalence of obesity was 23% in both men and women. Level of physical activity was mostly low or very low. BMI was inversely associated with consumption of pulses, rotisserie food, chocolate, sweets and physical activity during leisure time and directly associated with consumption of meat. Mean value of total cardiovascular risk was 4% in women and 11% in men. One percent of women and 16% of men were at high cardiovascular risk (≥ 20% in 10 years). Normal weight persons were four times more likely to be at low risk than obese persons.

**Conclusion:**

This study demonstrated the feasibility of a surveillance network of GPs in Italy focusing on obesity and other CVD risk factors. It also provided information on lifestyle habits, such as diet and physical activity.

## Background

Over the last decades, a slow but steady decrease can be observed in cardiovascular disease (CVD) mortality in industrialized countries. This is partly due to improvements in pharmacological treatment during both acute and chronic phases, reduction in levels of established major risk factors – total cholesterol and hypertension –, better pharmacological control of risk factors, and reduction in smoking habit [1,2]. An inverse trend can be observed for obesity, whose prevalence is steadily increasing [3].

Scientific evidence accumulated over recent years has prompted the American Heart Association to identify obesity as the major and modifiable risk factor for CVD, the UK Government (Department of Health) to include obesity as one of the six key priorities in the White Paper *Choosing Health: Making Healthy Choices Easier *and the Italian Ministry of Health to launch the Project *'Guadagnare Salute' *which foresees multidisciplinary interventions in the field of diet and physical activity [4-6]. Promotion of physical activity and healthy diet are regarded as key factors to prevent and reduce overweight and obesity epidemic. In USA, the Healthy People 2010 programme identifies physical activity as one of the public health top priorities [7]. The Public Health Programme 2003–2008 of the European Union invites countries to implement initiatives that sustain and promote physical activity [8]. In 2005, the Aprifel, the French agency for research and information on fresh fruits and vegetables, the Italian Ministry of Health and the National Centre for Disease Prevention and Control (CCM) organized the International Conference on Health Benefits of Mediterranean Style Diet – From Scientific Evidence to Health Prevention Actions with the aim of highlighting the latest scientific knowledge on health benefits of Mediterranean style diet and the strategic perspectives for health operators [9].

In Italy, a national surveillance network for overweight, obesity and other cardiovascular risk factors does not exist. Presently, latest available data on overweight and obesity come from the multi-scopes survey performed in 2005 and published in 2007 by the Italian Institute of National Statistics (ISTAT), collecting self-reported information on health status [10], and from the Epidemiologic Cardiovascular Observatory (OEC) of the Progetto CUORE, estimating prevalence of overweight, obesity and other CVD risk factors in about 10,000 Italian men and women aged 35–74 years examined between 1998 and 2002. [11]. Many initiatives are taken at the regional level, but they are mainly targeted to children and adolescents [12-16]. Therefore, there is a pressing need to establish a national surveillance system for collecting reliable and comparable data and implementing prevention procedures at individual and population levels.

Following the experience of other Italian studies trying to evaluate the feasibility of a surveillance network for other risk factors and diseases (alcohol, smoking, physical inactivity, renal diseases) [17-20], an Italian pilot project was implemented in January 2006 with the main aim of evaluating the feasibility of a surveillance network for CVD and obesity. Secondary aims were to assess the prevalence of cardiovascular risk conditions and distribution of overweight and obesity in men and women aged 35–74 years, to describe lifestyle habits such as diet and physical activity, and to investigate the association between lifestyle, obesity and predicted cardiovascular risk in the general population.

The study lasted about two years and involved general practitioners (GPs) to perform screening examinations to assess cardiovascular risk, lifestyles habits (physical activity, diet and smoking) and drug use among their patients. Particular focus was laid on overweight and obese persons.

## Methods

### Screening

Forty GPs from the Italian Association of General Practitioners (SIMG) and homogeneously distributed across the country were engaged. To ensure uniformity in the application of screening procedures, a training course for GPs on standardization of measurements was organized.

**Figure 1:**
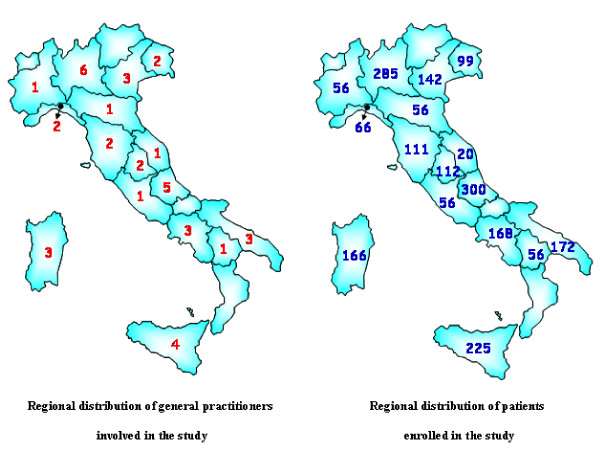
Geographical distribution of general practitioners and patients involved in the study.

At least one GP per region participated in the study [Figure 1]. Each participating GP had the task to screen 56 patients aged 35–74 years, randomly selected by the National Institute of Health (ISS) from each GP's patient list in order to have 7 patients in each age decade and sex (35–44, 45–54, 55–64, 65–74 years). Selected patients were invited by letter to the ambulatory of their own GP.

The screening protocol foresaw the completion of a questionnaire containing questions on anagraphical position, such as marital status and educational level, habits and lifestyles, remote pathological anamnesis (angina pectoris, myocardial infarction, cerebrovascular accidents, TIA, intermittent claudication), current drug treatments (antihypertensives, lipid-lowering, oral antidiabetics), family history (father, mother, sisters/brothers) of premature coronary heart disease, cerebrovascular accidents and family history of diabetes. Anthropometric (weight, height, waist circumference, hip circumference) and blood pressure measurements were performed using standardized methodologies (standard electronic scale, wall height ruler, tape measure, mercury sphygmomanometer). Laboratory analyses considered for risk assessment were: total cholesterol (TC), HDL-cholesterol (HDL-C) and glycaemia. Total cardiovascular risk (TCR) was assessed using the CUORE Project risk function [21,22].

A data entry software intended for GPs was developed to facilitate the process of data collection and storage during the examination of patients. At the end of the study, every GP sent collected data to the ISS for processing. Data sent by the 40 GPs referred to 1,044 men and 1,046 women, that is an average of 55 patients per GP. Written information consent was obtained from each study participant.

### Quality controls

The software provided to GPs for data entry offers automatic checks for variables congruence (e.g. an information related to the number of daily smoked cigarettes could not refer to a non-smoker) and alerts GPs to missing data.

The main aim of this pilot study was to assess the feasibility of a network of GPs for CVD surveillance, prevention and health promotion. For this reason, collected information were carefully evaluated for completeness and reliability.

For completeness analysis, the percentage of missing values for each variable was calculated; for reliability analysis, anthropometric (height, weight, waist and hip circumference) and blood pressure measurements were considered: the distribution of the terminal digit of recorded blood pressure readings and the percentage of equal consecutive readings were calculated [23]. For height, the percentage of terminal zeros was calculated as well; for weight, waist and hip circumferences the terminal decimal and whole digits were considered. Homogeneity of terminal digit distribution indicates a correct application of standard methodologies for risk factors measurements.

### Data analysis

Data on 2,090 patients included in the lists of 40 GPs were transmitted to the ISS.

Demographic characteristics (age, educational level and marital status) and the adverse conditions (moderate or low physical activity at work and during leisure time, smoking habit, obesity, hypertension, hypercholesterolemia and pharmacological treatment) were reported as prevalence. Definitions used in the data analysis of the OEC for hypertension, hypercholesterolemia and diabetes were taken into consideration to make results comparable [11]. The distribution of overweight and obesity was calculated considering the following categories: normal weight as body mass index (BMI) 18.5–24.9 kg/m^2^, overweight as BMI 25.0–29.9 kg/m^2 ^and obesity as BMI ≥ 30.0 kg/m^2 ^or waist circumference > 88 cm in women and > 102 cm in men. If not differently indicated, results and comments hereinafter refer to BMI.

Mean values were calculated for continuous major cardiovascular risk factors and consumption of some foods. The frequency of food consumption per week was recorded for the following food items: meat, cheese, fish, pulses, chocolate, fried food, sweet, wholemeal food, rotisserie food and sugar drink. Daily consumption was asked for fruits and vegetables. Alcohol intake was recorded as grams per day.

The relation of BMI and other CVD risk factors to consumption of some food items, alcohol intake and physical activity was assessed by univariate and multivariate analyses. For univariate analysis, age-adjusted Pearson's correlation coefficients (r) were calculated to assess relations between couple of variables; for multivariate analysis, multivariate linear regression models were performed considering CVD risk factors as dependent variables and consumption of food items, alcohol intake and physical activity as independent covariates (all models were also age and sex adjusted). In order to compare the effect of independent covariates, standardized coefficients of regression models were calculated (regression coefficient multiplied by the ratio of the standard deviation of the independent variable to the standard deviation of the dependent variable). Only significant coefficients were reported.

The 10-year TCR was assessed by GPs through the data entry software using the individual risk score built within the longitudinal study of the Italian CUORE Project [22]. This score allows to estimate the probability of experiencing a first cardiovascular event (myocardial infarction, stroke) over the next 10 years knowing the level of eight risk factors for CVD (age, gender, SBP, TC, HDL-C, diabetes, smoking habit and use of antihypertensive medication).

The TCR was assessed by GPs on 45% of those patients considered eligible for application of the CUORE Project risk function (complete information for the risk factors included in the CVD risk assessment, risk factors values within the reference limits, age range 35–69 years, free from previous cardiovascular events). For the remaining 55%, TCR was assessed within the present analysis using the risk factors values collected by GPs.

On the basis of the TCR value, patients were classified as low risk (TCR < 3.0%), risk to be kept under control through the adoption of a healthy lifestyle (TCR 3.0–19.9%), high risk (TCR ≥ 20.0%). Age-adjusted ANOVA models were used to assess mean difference between normal weight and obese, and the relation between BMI (or waist and hip circumferences) and TCR.

After checking for homogeneity of variances, comparisons among mean values of men and women were made using the t-test. In absence of homogeneity, the Welch test was used. The independence between categorical variables was assessed using the chi-square test.

Results associated to a p-value ≤ 0.05 were considered statistically significant. Quality controls and statistical analyses were performed using SAS software version 8.2 (SAS Institute, Cary, NC).

## Results

### Quality control

As the age distribution between men and women was similar, age adjustments were not needed to compare results among sexes.

The completeness of collected information was considered satisfactory. Percentages of recorded information are the following: 98% for blood pressure measurements; 94% for laboratory analyses such as TC, HDL-C and glycaemia; 98% for anthropometric measurements. Through the questionnaire, smoking habit was registered for 98% of sample, physical activity at work and during leisure time for 70% and 98% respectively, and food consumption frequency for 98%; information on medications use were available for 95% of sample.

The distribution of the terminal whole digit of blood pressure readings was found to be highly concentred: more than 50% of readings had zero as terminal digit. The proportion of equal readings between first and second measurements was 50% for SBP and 40% for DBP. Also for anthropometric measurements, the distributions of the terminal whole and decimal digits were concentrated on zero.

### Sample characteristics and risk conditions

Table 1 shows the main sample characteristics: more than 70% of women and 80% of men were married or cohabitant; 41% of women and 42% of men had high school diploma or university degree (Table 1). Ninety percent of women and 78% of men declared to make low or very low physical activity at work (mostly sedentary work or involving standing/walking) (Table 1), 10% of women and 22% of men declared to make high or very high physical activity at work (work involving much walking, manual work involving strenuous efforts). Physical activity during leisure time was low or very low (sedentary activity or moderate physical activity for no more than 4 hours a week) for both men and women (Table 1); only 5% of women and 11% of men declared to make high or very high physical activity during leisure time (enjoying sport as a hobby, doing heavy gardening, or exercising regularly for agonistic activity).

**Table 1:** Demographic characteristics and conditions at risk.

	**WOMEN**		**MEN**	
	**N**	**%**	**N**	**%**
***Demographic characteristics***				
				
**Age classes**				
35–44	247	24	248	24
45–54	260	25	250	24
55–64	265	25	261	25
65–74	274	26	285	27
				
**Marital status**				
Single	116	11	123	12
Married or live-in-partner	770	74	867	83
Separated or divorced	50	5	37	4
Widowed	110	11	15	1
				
**Education**				
College or university	113	11	131	13
High school	306	30	298	29
Secondary school	335	33	404	39
Primary school	275	27	200	19
				
***Conditions at risk***				
				
**Physical activity at work**				
Very low	261	33	250	38
Low	451	57	262	40
				
**Physical activity during leisure time**				
Very low	562	55	440	43
Low	402	40	474	46
				
**Body Mass Index, kg/m**^2^				
25.0 – 29.9	348	34	512	50
> = 30.0	234	23	235	23
				
**Waist circumference, cm**				
> 88 for women, > 102 for men	458	45	283	28
				
**Smoking habit**	183	18	269	26
Hypertension*	400	39	427	42
**Antihypertensive medication**	365	37	378	38
Hypercholesterolaemia**	332	33	326	32
**Lipid-lowering medication**	117	12	156	16
Diabetes***	98	10	137	14
**Diabetes specific medication**	67	7	97	10

Men and women 35–74 years.

*Systolic blood pressure ≥ 160 mmHg or diastolic blood pressure ≥ 95 mmHg or use of antihypertensive medications.

** Total cholesterol ≥ 6.2 mmol/l or use of lipid lowering medications.

***Glycemia ≥ 7 mmol/l or use of specific medications for diabetes.

Prevalence of overweight was 34% in women and 50% in men; 23% of both men and women were obese. Waist circumference exceeded the threshold for obesity in 45% of women and 28% of men. Forty one percent of women and 27% of men were normal weight. Mean value of BMI fell in the overweight category for both men and women (Table 2).

**Table 2:** Mean and standard deviation of blood pressure, blood test and anthropometric measurements.

	**WOMEN**			**MEN**		
	**N**	**mean**	**standard deviation**	**N**	**mean**	**standard deviation**
**Systolic blood pressure, mmHg**	1,016	131.9	17.4	1,027	133.5	16.0
**Diastolic blood pressure, mmHg**	1,016	79.4	8.9	1,027	81.1	9.2
**Serum Total Cholesterol, mmol/l**	978	5.5	1.0	984	5.3	1.0
**Serum HDL-Cholesterol, mmol/l**	978	1.5	0.4	980	1.3	0.3
**Glycaemia, mmol/l**	978	5.4	1.3	984	5.8	1.6
**Weight, kg**	1,016	67.5	12.7	1,027	80.7	13.0
**Height, cm**	1,016	159.2	6.9	1,027	171.2	7.2
**BMI, kg/m**^2^	1,016	26.7	5.3	1,027	27.5	4.2
**Waist circumference, cm**	1,016	87.8	13.0	1,026	96.8	11.4
**Hip circumference, cm**	1,002	99.0	12.3	1,007	100.9	11.2

Men and women 35–74 years.

### Dietary and physical activity habits

Data on dietary habits collected through administered questionnaire showed that women ate more wholemeal food, vegetables and chocolate compared to men; men had a higher weekly consumption of alcohol, meat, fried food and sugar drinks than women (Table 3). Normal weight persons declared to have a higher average number of meals eaten away from home per week compared to obese persons. No statistically significant differences were found between normal weight and obese persons with regard to frequency of breakfast consumption away from home. Similar results were recorded using waist circumference as obesity indicator. Eight percent of normal weight women and 0.4% of obese women declared to make high or very high physical activity during leisure time; among men, 15% and 8% respectively.

**Table 3:** Food frequency per week and alcohol intake.

	**WOMEN**				**MEN**			
	**Median**	**Mean**	**Min**	**Max**	**Median**	**Mean**	**Min**	**Max**
Meat *	3	3.4	0	8	3	3.7	0	8
**Cheese**	3	3.3	0	8	3	3.4	0	8
**Fish**	2	1.7	0	8	2	1.8	0	8
**Pulses**	2	2.2	0	8	2	2.1	0	8
Chocolate *	1	2.3	0	8	1	1.9	0	8
Fry food *	1	0.8	0	7	1	1.0	0	8
**Sweet**	1	1.6	0	8	1	1.7	0	8
Wholmeal food *	0	1.9	0	8	0	1.5	0	8
**Rotisserie food**	0	0.7	0	8	0	0.8	0	7
Sugar drink *	0	0.9	0	8	0	1.1	0	8
**Fruits**	2	2.5	0	8	2	2.4	0	8
Vegetables *	2	1.9	0	8	1	1.7	0	8
Alcohol intake*	0	8.1	0	83	20	24.6	0	239

Men and women aged 35–74 years.

Fruits and vegetables food frequency per day. Alcohol intake expressed in grams.

* Significant difference between means of men and women (t-test; p-value ≤ 0.05).

Considering food frequency, alcohol intake and physical activity singly, BMI was found to be significantly and positively correlated, although not strongly, with meat consumption and alcohol intake (r = 0.05 and r = 0.08 respectively), while significant negative correlations were found between BMI and consumption of fish (r = -0.05), pulses (r = -0.11), wholemeal food (r = -0.05), vegetables (r = -0.04), chocolate (r = -0.07), sweets (r = -0.05), rotisserie food (r = -0.05) and level of physical activity during leisure time (r = -0.19). Correlation of BMI to physical activity at work and other food items consumption was not statistically significant. Similar results were recorded using waist circumference as obesity indicator.

Multivariate regression model confirmed that higher consumption of meat was associated with higher levels of BMI, while higher consumption of pulses, chocolate, sweets and rotisserie food was associated with lower levels of BMI (Table 4). Physical activity during leisure time had the strongest inverse impact on BMI (Table 4). Physical activity during leisure time was found to be statistically associated with all risk factors considered with the exception of TC (Table 4); no association was found with physical activity at work.

**Table 4:** Relation between food frequency, physical activity, alcohol intake and main cardiovascular risk factors.

	**SBP**	**DBP**	**TC**	**HDL-C**	**Glycemia**	**BMI**	**Waist**
	**Std Coeff**	**Std Coeff**	**Std Coeff**	**Std Coeff**	**Std Coeff**	**Std Coeff**	**Std Coeff**
**Meat**			0.061*			0.052*	0.051*
**Cheese**							
**Fish**				-0.063*			
**Pulses**	-0.051*					-0.074**	
**Chocolate**				0.122***	-0.050*	-0.071**	-0.077**
**Fry food**				-0.057*			0.056*
**Sweet**	-0.071**					-0.056*	-0.055*
**Wholmeal food**	-0.065**						
**Rotisserie food**				-0.058*		-0.072**	
**Sugar drink**				-0.075**			0.052*
**Fruits**				-0.078**			
**Vegetables**				0.062*			
**Physical activity at work**							
**Leisure-time physical activity**	-0.049*	-0.074**		0.132***	-0.066**	-0.187***	-0.195***
**Alcohol intake **	0.083**	0.073**	0.146***	0.064*	0.078**		

Men and women aged 35–74 years.

Variables on the top of the columns are considered as dependent variables. Food intake, physical activity and alcohol intake are considered as independent covariates in each model. The seven models are also age and sex adjusted.

Std Coeff is the standardized coefficient of multivariate linear regression model.

Significance: *** p < 0.001, ** p < 0.01, * p < 0.05.

All food frequencies are per week except for fruits and vegetables (per day). Alcohol intake expressed in grams.

SBP: systolic blood pressure (mmHg); DBP: diastolic blood pressure (mmHg); HDL-C: HDL-cholesterol (mmol/l); glycaemia: fasting plasma glucose (mmol/l); BMI: body mass index (kg/m^2^); waist: waist circumference (cm).

### Total cardiovascular risk

Three percent of sample (48 men and 10 women) declared to have experienced a cerebrovascular or coronary event before entering the study. The TCR was calculated for 806 women and 805 men. Mean value of TCR was 4% in women (standard deviation was 5%) and 11% in men (standard deviation was 11%). One percent of women and 16% of men were at high cardiovascular risk (TCR ≥ 20.0% in 10 years).

In both men and women the mean value of TCR was associated with BMI classes (women: 3%, 5%, 7%; men: 8%, 11%, 13% for normal weight, overweight and obese respectively); similar results were recorded considering waist circumference (women: 3% and 6% for non-obese and obese respectively; men: 9% and 14%).

**Figure 2:**
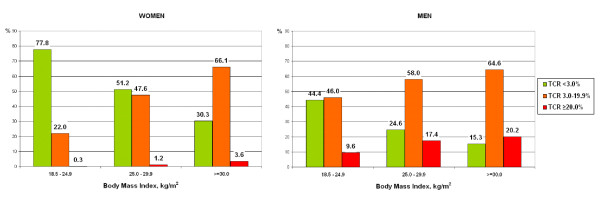
**Distribution of Total Cardiovascular Risk (TCR) by Body Mass Index categories**. Men and women 35–69 years without previous cardiovascular disease.

Thirty percent of obese women versus 78% of normal weight women, and 15% of obese men versus 44% of normal weight men were at low cardiovascular risk (Figure 2). Four percent of obese women versus 0.3% of normal weight women, and 20% of obese men versus 10% of normal weight men were at high cardiovascular risk.

## Discussion

Obesity represents a threat to health which is peculiar, although not exclusive, to the so-called 'well-being societies'. A constant increase in the availability of food on one side and an increasingly sedentary lifestyle on the other side appear to be the main factors behind this situation. Many degenerative diseases are associated with obesity: diabetes, hypertension, ischemic heart disease, heart failure, some types of cancers (endometrial, colorectal, kidney, gall bladder and breast cancer in post-menopausal women), and osteoarticular pathologies. Obesity has a rather complicated association with cardiovascular risk: some researchers argue that this association is indirect and dependent on an increased prevalence of diabetes, hypertension and dysplipidemia, while others consider obesity as an independent risk factor for CVD [24,25].

All things considered, initiatives aiming at monitoring trends in national obesity and promoting prevention actions at local level are of strategic importance. In 1984, the Center for Disease Control and Prevention (CDC) of USA developed the Behavioral Risk Factor Surveillance System to collect monthly in all 50 states information on health risk behaviours and preventive health practices [26]. In Europe, the European Physical Activity Surveillance System (EUPASS) has been implemented to contribute to the establishment of an appropriate, cost-effective and feasible Community health monitoring system to be achieved by developing and testing a surveillance system, using physical activity, being a major indicator of public health, as its substantive focus and starting point [27]. In UK, the Counterweight programme developed and tested a weight management intervention programme in primary care [28,29].

Italy still lacks a valid national system of surveillance to track overweight, obesity and other cardiovascular risk factors. For this reason, a pilot project was implemented with the aim of evaluating the feasibility of a national surveillance network for obesity and other cardiovascular risk factors. Forty GPs were engaged to collect their patients' data on dietary habits, physical activity, use of medications, smoking habit, major cardiovascular risk factors level and anthropometric measurements using standardized methodologies.

The completeness of collected information on routinely performed measurements and laboratory assays, as well as information collected through questionnaire such as dietary habits, physical activity level and smoking habit was considered satisfactory. Younger persons were easy to enrol, in fact the homogeneity in age distribution foreseen in the protocol was not altered. The parameters used for the analysis of reliability of collected data referred to quality controls performed in the epidemiological field, where the accuracy of measurements represents a crucial bond for reliability of estimates [23]. In this study, the analyses on the distribution of terminal digit of blood pressure readings and anthropometric measurements represented a valid self-monitoring tool for GPs using standardized methodologies. However, a good reliability of collected information was confirmed by the significance found in known associations (dietary habits, physical activity, BMI, waist circumference, cardiovascular risk factors) and by homogeneity of results with those obtained through OEC survey [11].

Given the availability of data, it was worth studying dietary habits, physical activity and relation of these with CVD risk factors, despite this went beyond the main aim of the study. Since cross-sectional design prevents determination of causality and assessment of lifestyle changes' effects (diet, physical activity) over time, analyses of data focused on association of cardiovascular risk factors with food frequency and overweight/obesity, underlying differences between men and women. Results showed that men had a greater tendency to be overweight compared to women, while obesity is a problem affecting both genders. Compared to men, women seemed to care more about their diet, eating healthy food, such as wholemeal food and vegetables, more frequently than did men. On the other hand, men ate food rich in saturated fats such as meat, fried food and sugar drinks more frequently than did women. Persons with lower BMI ate sweets, rotisserie food and chocolate more frequently than did overweight persons and declared to eat away from home more frequently. This behaviour may indicate either that persons with high BMI or waist circumference knew their weight problems and were willing to solve them or that persons overweight/obese had the tendency to not declare their unhealthy dietary habits. Although fish, wholemeal food and vegetables consumption resulted not significant when adjusted for all other foods consumption, a more frequent consumption of these food items was found to be correlated with persons having a lower BMI or waist circumference. Physical activity during leisure time was a better discriminator than physical activity at work for assessing the tendency to overweight or obesity; however, both types of physical activity were low or very low. Men made more high or very high physical activity during leisure time compared to women. Alcohol intake was found to be associated with most cardiovascular risk factors and, together with physical activity during leisure time, was one of the most important factors within the multivariate analyses. Alcohol consumption was higher for men than for women.

The level of 10-year TCR calculated through the CUORE Project function ranged in the risk category to be kept under control through the adoption of healthy lifestyle (risk category between 3.0% and 19.9%) for both men and women. The likelihood for normal weight persons to be low risk was almost four times higher compared to obese persons.

It is not easy to establish to what extent patients included in this study are representative of the Italian patient population. As for cardiovascular risk factors comparison, mean levels of risk factors and prevalence of risk conditions were in line with results obtained through OEC survey: mean BMI, SBP, HDL-C values were quite similar to those reported by OEC (BMI 26 kg/m^2 ^for women and 27 kg/m^2 ^for men, SBP 135 mmHg for women and 132 mmHg for men, HDL-C 1.5 mmol/l for women and 1.3 mmol/l for men). Mean values of TC, glycemia, hypertension and hypercholesterolemia were higher for both genders (OEC values: TC 5.5 mmol/l for women and 5.3 mmol/l for men, glycaemia 4.8 mmol/l for women and 5.2 mmol/l for men, hypertension 31% for women and 33% for men, hypercholesterolemia 25% for women and 21% for men) [11]. The opposite was found for smoking habit: OEC showed higher prevalence compared to this study (OEC values: 21% for women and 30% for men). Physical inactivity prevalence was found to be higher in this study, especially for women. Differences could be ascribed to the different period of screening (OEC survey was performed at the end of 1990s); this is particularly true for smoking prevalence, which has been changing after the introduction of the Italian anti-smoking legislation in January 2005 banning smoking in public places.

Overall, the picture that emerged from this pilot project is positive, showing a population with mostly healthy, but certainly improvable, dietary habits (mean consumption of meat 3/4 times per week, fish and pulses twice per week, two portions of vegetables and fruits per day, small amount of fried food, acceptable mean level of alcohol intake per day) and a mean cardiovascular risk level not exceeding the threshold of high risk.

Obesity prevalence should not be underestimated and is probably due to food quantity rather than food quality, and to a mostly sedentary lifestyle. Monitoring body weight and risk factors levels represent a first step towards persuading the general population to implement preventive actions against the onset of diseases leading to worsening quality of life or even reduction in the years of life. Obesity has its roots in the common way of thinking, which must be changed through lifestyle interventions targeted to the general population.

Little rigorous research study exist on how effective training is in helping primary care teams to manage obesity more effectively [30]; nevertheless, several studies showed that GPs have a significant role to play in preventing and diagnosing weight problems and in improving initial counselling [31,32]. It is a fact that they are often consulted [33] and most patients believe that their GPs could help them lose weight [34]. A cross sectional telephone survey conducted in 2002 in France on 600 GPs reported that most of them regarded obesity as a disease and agreed that their role includes weight problem management but complain lack of time as a frequent problem [35], which is confirmed also by other studies [36,37], and generally known to be a significant barrier to preventive care in general practice [38,39]. In our pilot project, a random selection of GPs was not feasible and it is now impossible to determine to what extent this choice influenced the study success. Preventing overweight and obesity could avoid further increase of health care use and related costs: Liset van Dijk et al. study [40] evaluated GPs consultations in moderate and severely overweight persons in Netherlands and reported that obese persons, regardless of different gender, age, social status and lifestyle, are more likely to consult their GP.

To ensure effective surveillance of prevention of obesity and CVD, the activity of GPs should be accompanied by other initiatives. As suggested by an increasing number of reports and as pointed out by Benjamin Caballero from The Johns Hopkins University also pointed out in his global epidemic of obesity overview, obesity is not only a disorder of individual behaviour but it is highly influenced by socioeconomic environment [41]. This is why prevention needs to take place simultaneously at the individual level and at the population level. Good social support can help give people the emotional and practical resources they need, particularly for coping with difficult life transitions.

The WHO Regional Office for Europe, within the *Strategy for the Prevention and Control of Noncommunicable Diseases*, on the occasion of the Copenhagen meeting held on 12 September 2006, indicated that effective interventions can include: control of the quality of consumer information about certain products, such as food, tobacco, alcohol; cooperation with industry through voluntary or enforced agreements to reduce levels of added salt, fat and sugars in manufactured foods; regulations within the built environment, for example through health impact assessment of transport and urban development proposals in order to promote walking and cycling [42]. The objectives of the strategy should be combined with integrated action on risk factors and their underlying determinants across sectors, with efforts to strengthen health systems toward improved prevention and control.

In Italy, beside population-based interventions implemented within the Project *'Guadagnare Salute'*, the CCM, in collaboration with ISS, has launched a national training plan aimed at increasing GPs' awareness about the key role of cardiovascular prevention, in order to implement an Italian national surveillance system following the objectives of the pilot study under consideration. The new skills acquired through the training course will translate into health objectives for the general population and for individuals: lifestyle changes among individuals at high risk and general population; reduction in the frequency of risk conditions and in the mean level of single risk factors (an exhaustive session is dedicated to overweight, obesity, nutrition and physical activity). The software cuore.exe, downloadable free of charge from the website of the CUORE Project , helps GPs to set up a data archive, to monitor cardiovascular risk and risk factors trends in patients over time, to keep a record of the prescribed therapies and lifestyle recommendations given to patients, to collect data related to obesity and overweight, and to print out the patient's cardiovascular risk score, including some recommendations for lifestyle and therapy, if prescribed. The Observatory of Cardiovascular Risk (ORC), coordinated by the ISS, pools data collected by GPs through the cuore.exe, supports GPs with quality control, and disseminate results for monitoring TCR and risk factors by genders, age and geographical area and evaluating the efficacy of prevention action in primary care. Preliminary data of the ORC are under evaluation.

## Conclusion

The active role of GPs in the process of prevention represents a potential key to success. GPs have the primary and complete responsibility toward their patients, contribute to the development of individual assistance plan and recommend specialist visits when necessary. This study has demonstrated the feasibility of a GPs surveillance network for obesity and CVD in Italy. It may support informative campaigns on how people can adopt healthy diet and increase daily physical activity; it also represents a good way to monitor overweight, obesity and CVD, and a great step toward assuring a good health in the future.

## Competing interests

The authors declare that they have no competing interests.

## Authors' contributions

CD: performed statistical analysis, interpretation of data and made substantial contribution in drafting the manuscript. CL: made substantial contribution to acquisition of data. OB: was involved in revising the manuscript critically. GR: was involved in revising the manuscript critically. PC: was involved in drafting the manuscript and making language revision. FD: made contribution to acquisition of data. LP: was involved in revising the manuscript critically. SG: made substantial contribution in conception and design, and revised the manuscript critically. All authors read and approved the final manuscript.

## Pre-publication history

The pre-publication history for this paper can be accessed here:


